# *Candida glabrata* Pneumonia in a Patient with Chronic Obstructive Pulmonary Disease

**DOI:** 10.1155/2016/4737321

**Published:** 2016-11-02

**Authors:** Onur Yazici, Mustafa Cortuk, Hasan Casim, Erdogan Cetinkaya, Ali Mert, Ali Ramazan Benli

**Affiliations:** ^1^Department of Chest Disease, Adnan Menderes University, Aydın, Turkey; ^2^Department of Chest Disease, Karabuk University, Karabuk, Turkey; ^3^Department of Chest Disease, Karabuk University Training and Research Hospital, Karabuk, Turkey; ^4^Department of Infectious Diseases, İstanbul Medipol University, İstanbul, Turkey; ^5^Department of Family Medicine, Karabuk University, Karabuk, Turkey

## Abstract

Pneumonia remains an important cause of morbidity and mortality among infectious diseases.* Streptococcus pneumoniae* and viruses are the most common cause of pneumonia. Candidiasis in such patients has been associated with haemodialysis, fungal colonization, exposure to broad-spectrum antibiotics, intensive care unit (ICU) hospitalization, and immunocompromised patients. The most common cause of infection is* C. albicans*. The case presented here is of a 66-year-old male patient diagnosed with* C. glabrata*. The patient suffered from chronic obstructive pulmonary disease.

## 1. Introduction

Pneumonia is a common respiratory tract disease and is one of the leading causes of mortality. Streptococcal and viral pneumonia are determined to be among the most common causes of community-acquired pneumonia in adults. In individuals with normal immunity,* Candida* species rarely cause pneumonia. The most common pathogen among the* Candida* species is* C. albicans*, particularly in subjects with reduced immunity or patients necessitating intensive care management [1].

*C. glabrata* is known as a nonpathogen* Candida* species.* C. glabrata* rarely acts as an infectious agent when compared to other* Candida* species and it is present within normal respiratory flora [2]. Few cases have been reported in which* C. glabrata* was determined to be an infectious agent causing pneumonia [3–6].

In this case report,* C. glabrata* pneumonia was diagnosed in a patient who has chronic obstructive pulmonary disease (COPD) and has been taking thyroid replacement therapy.

## 2. Case Presentation

**Figure 1:**
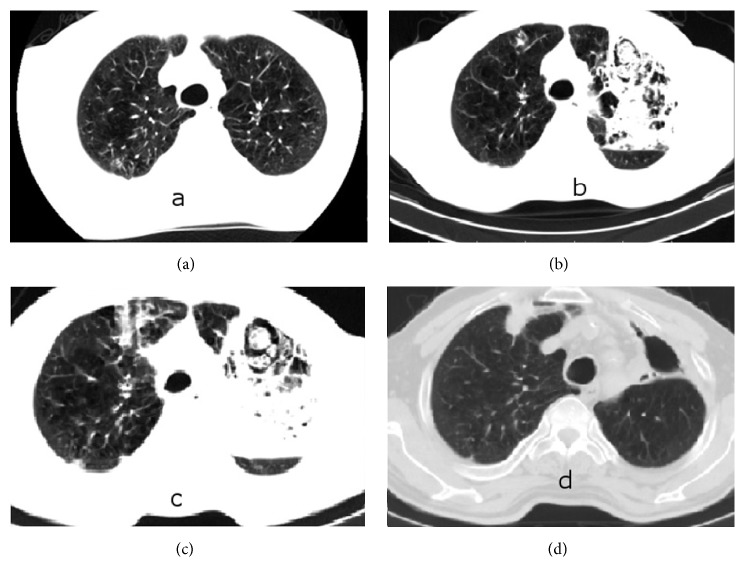
Chest tomography views on 4th day (a), 18th day (b and c), and 6th month (d) of hospitalization.

**Figure 2:**
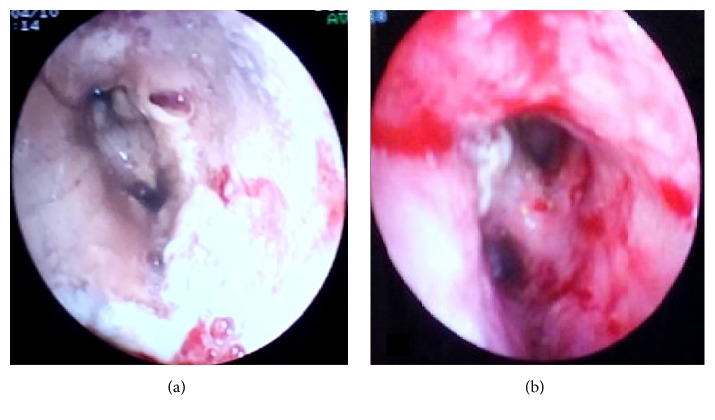
The appearance of the left main bronchus in the bronchoscopic examination performed before (a) and after (b) the 15th day of voriconazole treatment.

A 66-year-old male patient was admitted with cough, purulent and blood-mixed sputum, and increased shortness of breath. His medical history included COPD for the last 5 years and total thyroidectomy performed 3 years ago with the diagnosis of multinodular goiter. When questioned about prescriptions, he was under inhaled formoterol, ipratropium bromide, and levothyroxine sodium, administered orally at 100 *μ*g/day. He had not been hospitalized in the last year. Physical examination revealed blood pressure of 110/70 mmHg and pulse of 89/minute; his temperature was 36.8°C and fingertip-measured oxygen saturation (SpO_2_) was 89% on room air. He presented with decreased breath sounds and prolongation of expiratory time. In addition, rales were present on auscultation of the mid and lower zones of the lungs. Laboratory investigation revealed C-reactive protein of 85 mg/dL, leukocytosis, with a white blood cell (WBC) count of 12.000/mm^3^, and increased erythrocyte sedimentation rate (35 mm/hour). The chest X-ray showed increased bronchovascular shadows of the lungs. Treatment of an inhaler bronchodilator, intravenous theophylline, and 40 mg/day methylprednisolone was started. An antibiotic combination of intravenous ampicillin sulbactam (4 g/day) and oral levofloxacin (500 mg/day) was administered. On the 4th day of treatment, the administered antibiotics were discontinued due to a deterioration in clinical features together with progression of infection parameters and cefoperazone sodium/sulbactam sodium combination (4 g/day) and moxifloxacin (400 mg/day) were started as new antibiotics. Computerized tomography (CT) of the chest was performed where emphysema and a small infiltration of right upper lobe posterior part were identified (Figure 1(a)). As the patient's clinical condition was getting worse and the infection parameters were not improving despite the treatment, a chest X-ray was obtained, which showed an increase of nonhomogeneous density on the left upper lobe. Additionally, flexible bronchoscopy was performed with normal findings except increased bronchial submucosal vascularity on the tenth day of being admitted to the hospital.* Streptococcus mitis* and* Candida* species were produced in the bronchoalveolar lavage obtained by left upper lobe. Therefore, the antibiotics were discontinued and linezolid 800 mg/day and fluconazole 200 mg/day were started intravenously. Meanwhile, an increase in blood sugar level was detected, which was considered to be related to intravenous corticosteroid. Therefore, the corticosteroid dosage was reduced and discontinued on the 12th day, after which the blood sugar level returned to normal. Since SpO_2_ continued to remain below 88% despite administration of 5 L/min oxygen and tachycardia and tachypnea were present, the patient was admitted to the intensive care unit and bilevel positive airway pressure (BPAP) treatment was applied. Despite this treatment, no improvement was observed in the patient. A further chest CT was obtained which showed a pneumonic appearance with patchy necrotic areas in the left upper lobe (Figures 1(b) and 1(c)). Flexible bronchoscopy was performed again on the 25th day of being admitted to the hospital and it revealed a view consistent with diffuse white* Candida* plaques in sites starting from the vocal cords and covering the entire trachea and both bronchial systems (Figure 2(a)). Bronchial biopsies and lavage of the left upper lobe were obtained. The bronchial biopsy revealed no fungus or similar pathology in the tissue. Because* Candida* spp. is reproduced in bronchoalveolar lavage taken at both 10th and 25th days, and* Candida* plaque is compatible with macroscopic appearance on bronchoscopy done at 25th day, it has been thought to be resistant* Candida* spp. and voriconazole treatment has been started. The bronchoalveolar lavage sample that* Candida* spp. reproduced on* Candida* plaques that are taken from BAL sample was sent to “Refik Saydam National Public Health Agency” for classification and antibiogram. Fluconazole resistant* C. glabrata* reproduced on the fungi culture that is done in this institution. The MIC90S value was reported as ≥32 *μ*g/mL for fluconazole and 1 *μ*g/mL and for voriconazole. We could not obtain* Candida* specific antibodies and mannan antigen tests that are used for the diagnosis of* Candida* infections, because it is not performed in our hospital. The fluconazole was discontinued and intravenous voriconazole 400 mg/day was started. Following this treatment, the clinical status of the patient gradually ameliorated, hemoptysis stopped, and infection parameters improved. The patient, who no longer required BPAP, was admitted to the clinical ward. Intravenous voriconazole was continued for 23 days. Control bronchoscopy performed on the 15th day of voriconazole treatment revealed almost complete recovery of the previously observed* Candida* plaques. Following significant improvement of his general status and dyspnea, the patient was discharged from the hospital with a prescription for oral voriconazole. Blood and urine cultures obtained during the hospitalization did not manifest any* Candida* growth. During ambulatory treatment with voriconazole,* E. coli* grew once in the urine culture and this was treated with ertapenem administered for 10 days. The voriconazole treatment was continued for 6 months. The appearance of air-fluid level at the left upper lobe and traction of the trachea to the left were identified during treatment. Left upper lobectomy was recommended because of this radiological appearance, but the patient refused surgery and so was continued to be followed up clinically and radiologically. The clinical situation of the patient did not worsen. Radiological and bronchoscopic recovery (Figure 2(b)) was seen on the last obtained CT (Figure 1(d)). Therefore, the idea of surgery was abandoned and the ambulatory follow-up of the patient currently continues uneventfully.

## 3. Discussion

*Candida* species exist as opportunistic pathogens in the microflora of the human body [7].* Candida* infections are encountered especially in patients hospitalized in intensive care units (ICU) and these infections prolong ICU stay and increase the mortality rate [8]. When* Candida* is produced, particularly in cases obtained from the respiratory tract, the most important problem is due to either colonization or invasive pulmonary candidiasis. Since* Candida* is an element of microflora, to differentiate from colonization, it is necessary to be identified in the tissue, in general.

Pulmonary fungal infections are being increasingly identified as infectious agents in immunocompromised subjects. In the article by Chen et al., in which pulmonary fungal infections were compiled,* C. glabrata* was reported in 4 out of 140 patients [9].

*Candida* pneumonia is caused either by candidemia via the hematogenous route or by aspiration from the oropharynx. Kobayashi et al. reported the case of a 71-year-old patient who was being fed through nasogastric catheter [3]. In this case, the probable contamination route was considered to be aspiration. Another 78-year-old patient, reported by Speletas et al., had chronic myeloid leukemia and was using imatinib mesylate [6]. In this patient, candidemia was not reported. Hamilton et al. reported a case of* Candida glabrata* pneumonia with candidemia in an immunocompetent patient [5]. Bankier et al. reported a case of* C. Glabrata* infection in a nonimmunocompromised patient [4]. To the best of our knowledge, no other case has been reported except those mentioned above.

Franquet et al. reported that there is no specific radiologic image on* Candida* pneumonia. It has been reported that, in the study, computerized tomography (CT) findings included multiple nodules and air-space consolidation and nodules surrounded by discrete areas of ground-glass opacity (CT halo sign) can be seen [10]. In our case consolidation and cavitation were also shown on the CT of left upper lobe and a nodule of right upper lobe. Although there are many respiratory signs and symptoms from the respiratory system on the admission day, the patients suffered from COPD for the last 5 years.

In the current case, the patient suffered from COPD and thyroid replacement therapy. There was no history of central catheter utilization or admission to hospital or intensive care unit. There was no history of vomiting and aspiration. Although the lower respiratory tract infection is a common complication of COPD that is being presented in the case, general immune deficiency has not been determined. Although colonization by* Candida* of multiple nonsterile sites has been declared, prolonged use of antibacterial antibiotics has been linked to increased risk of invasive candidiasis [11]. In our case,* Candida* plaques were seen under the vocal cords which were excepted as sterile normally. Also, on the laboratory parameter, CRP sedimentation and WBC were found to be high on the day when the patient is hospitalized and these parameters got worse despite the antibiotics. The status of the patient deteriorated from the beginning and no response was observed from the administered nonspecific treatments. Since the second bronchoscopy, which was performed while the patient was under empirical fluconazole treatment, revealed* Candida* plaques in the entire bronchial system starting from the vocal cords, the fluconazole treatment was discontinued before the culture results were obtained and voriconazole was started. Thereafter, the culture result was received as* C. glabrata* and its resistance against fluconazole was verified by antibiogram. Previous publications have frequently identified the resistance of* C. glabrata* to azole group antifungals [12].

To differentiate a pulmonary infection caused by* Candida* from colonization, the microbiological agent should be identified in tissue, in general. In the current case, we showed wide* Candida* plaque on the bronchoscopy, but, unfortunately, we could not identify it microscopically in the tissue. Nevertheless* C. glabrata* were identified by bronchoalveolar lavage twice. Although* Aspergillus* type fungi were intensively sought in both the biopsy specimens and the cultures, due to a similar radiological appearance, they were not identified.

## 4. Conclusion

*C. glabrata* is quite a rare cause of pneumonia. Although it is quite rare, it may also be the cause in ambulatory patients. It should be kept in mind especially in cases where the clinical condition does not improve despite treatment with azole group antifungal agents and* Candida* species are detected in respiratory tract isolates. It should also be kept in mind that successful treatment is possible with voriconazole.
